# Subtrochanteric fracture in previously treated breast cancer patient handled by proximal femoral nail: A case report

**DOI:** 10.1016/j.ijscr.2023.108411

**Published:** 2023-06-21

**Authors:** Mohamad Ali Farho, Mohamad Nabhan Sawas, Maen Alnajjar, Mohammed Al-Mahdi Al-Kurdi, Ahmad Nawlo, Hani Alloush

**Affiliations:** a Faculty of Medicine, University of Aleppo, Aleppo, Syria; b CME Office, Faculty of Medicine, University of Aleppo, Aleppo, Syria; c Department of Orthopaedic Surgery, Faculty of Medicine, Aleppo University Hospital, University of Aleppo, Aleppo, Syria

**Keywords:** Breast cancer, Breast invasive ductal carcinoma, Bone metastases, Subtrochanteric femoral fracture, Proximal femoral nail, Mirel's scoring

## Abstract

**Introduction:**

Breast cancer (BC) is the most common and high mortality rate cancer in females. The main complication of BC is metastases, where bone metastases (BM) are present in 90 % of women with distant metastases and commonly recurrence after BC therapy. However, treatment options are numerous, and improving patients' quality of life (QoL) is a priority.

**Presentation of case:**

A 58-year-old female patient presented to the emergency department with pain and movement restriction in the right lower extremity after minor trauma. Clinical history included a surgically resected BC eight years ago, besides chemotherapy and radiotherapy. After clinical and radiographic examination, we encountered a subtrochanteric femoral fracture although the patient is in the end stage, the multidisciplinary team discussed the surgery option with the patient and eventually internally fixed the fracture.

**Discussion:**

Subtrochanteric femur fractures represent a challenging orthopedic issue, ranging from 10 % to 34 % of all hip fractures. Hence, after a detailed discussion, the proximal femoral nail (PFN) was the procedure of choice acording to the patient's preferences and tumor prognosis. Proximal femoral metastasis treatment aims to improve the quality of life (QoL), alleviate bone pain, and rehabilitate skeletal function.

**Conclusion:**

In this case report, we highlight the surgical decision consequences for a patient with end-stage cancer, as it may put their life at risk or improve their QoL, likewise the patient in this report.

## Highlights

• PFN was the procedure of choice depending on the patient's preferences and tumor prognosis.
• Subtrochanteric femur fractures represent a challenging orthopedic issue.
• Improving the outcome in BC by diagnosing and treating BM prior to the onset of significant functional deficits
• It's crucial for orthopedic surgeons to be involved at an early stage and not just when patients have pathological fractures.

## 1 Introduction

Breast cancer (BC) is a common malignancy diagnosed in females and is considered a prime cause of death among women globally [1,2]. Mortality is related to distant metastases formed mainly in the liver, bones, and lungs. Nearly 5 to 8 % of diagnosed patients have distant metastases [3]. Moreover, bone metastases (BM) are present in 90 % of women with distant metastases and commonly recurrence after BC therapy [4]. The main sites of BM include the vertebral column, the hip bone, and the proximal femur. However, the proximal femur has several locations for metastases formation; the neck, subtrochanteric, and intertrochanteric sites [5]. These lesions result in bone pain and pathological fractures. Survival in BM patients may reach four years. Hence, early diagnosis and treatment relieve the patients' outcomes. Proximal femoral metastasis treatment aims to improve the quality of life (QoL), alleviate bone pain, and rehabilitate skeletal function [6]. A cephalomedullary device (internal fixation) is the option in subtrochanteric fractures once the femoral head and neck can reinforce the implant [5,7]. Besides, the proximal femoral nail (PFN) is an appropriate procedure as it reduces surgical dissection, bleeding, trauma, and postoperative complications [8]. In this case, we report a subtrochanteric metastatic fracture fixed using PFN in a previously treated BC woman. This manuscript was prepared by the SCARE 2020 guidelines [9].

## 2 Case presentations

A 58-year-old woman presented to the emergency department with severe pain and movement restriction in the right hip joint. Medical history revealed that she had been diagnosed with breast invasive ductal carcinoma (IDC) eight years ago and successfully treated with modified radical mastectomy followed by chemoradiotherapy. She also complained of extensive bone pain in the last couple of months, which was uncontrolled by analgesics.

**Fig. 1:**
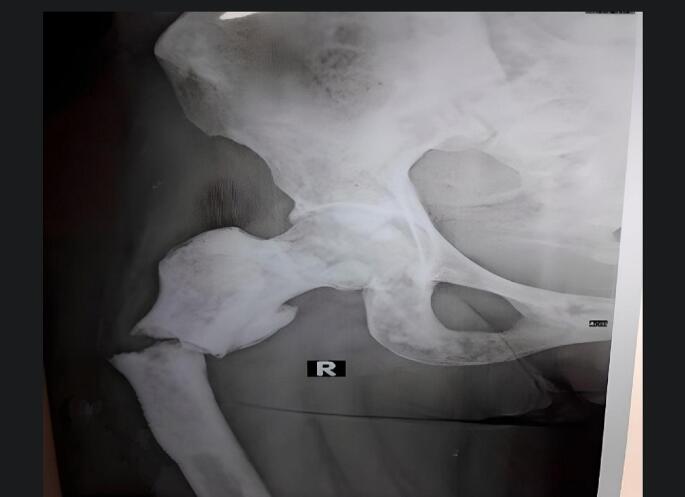
Femur X-Ray shows right transverse subtrochanteric fracture.

**Fig. 2:**
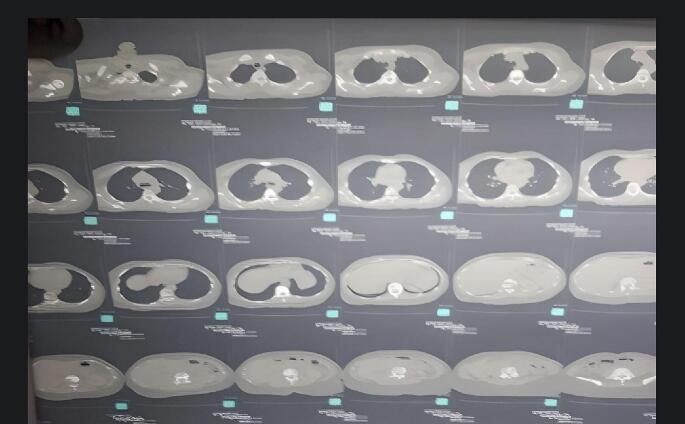
ECCT scan of the pelvic.

**Fig. 3:**
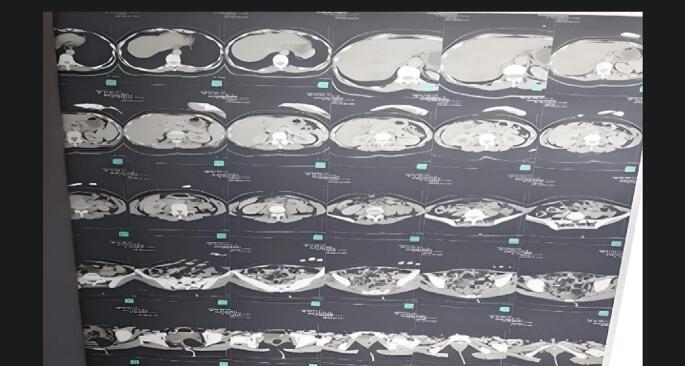
ECCT scan of the chest, and abdomen.

**Fig. 4:**
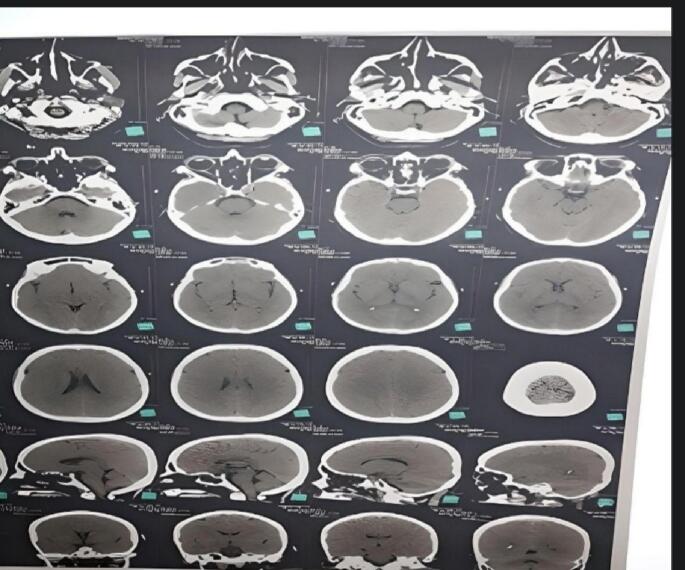
Brain CT scan shows normal tissue.

The femur X-ray showed a transverse subtrochanteric femoral fracture (SFF) (Fig. 1). Also, the chest, abdomen, and pelvis enhanced-contrast computed tomography (CT) scan demonstrated osteolytic and sclerotic lesions with a displaced fracture in the right femoral neck (Fig. 2). The liver was normal-sized with homogeneously enhanced lesions, the largest of which was (35 mm) in the right hepatic lope without cholangiectasis or intrahepatic veins dilation (Fig. 3*).* The CT scan also excluded ascites, pleural and pericardial effusion, free fluids in the pelvis, and lymphadenopathy in the axillary fossa, chest, abdomen, or pelvis (Fig. 3). Fortunately, the brain CT scan showed normal findings (Fig. 4), while we imputed the pathological fracture to metastasis, according to the radiographic findings and clinical history A multidisciplinary team of orthopedics surgeons, radiologists, and oncologists discussed available treatment options with the patient and agreed on PFN surgery, considering the chief complaint, other systems reviews, radiographic findings, medical history, survival rate, and QoL improvement.

We administered the patient antibiotics before surgery that she underwent on the day of admission under general anesthesia by a consultant orthopedic and three assistants. The surgery lasted for 1 h and a half in a supine position on a fracture table. Besides, surgeons stated difficulty in nail insertion due to the sclerosing metastatic lesions in the femur. We reported no postoperative complications and the patient was discharged a day after the surgery. We radiographically identified the success of surgery, with a regular eight monthly follow-up by visits and phone reassuring. The patient is now well-rehabilitated and satisfied after improving her skeletal ability and QoL and in the meantime, the patient does not suffer from any bone pain, despite the metastatic lesions and end-stage condition.

## 3 Discussion

BC is the top cause of cancer-associated mortality among women globally [2]. The major reason for pain among cancer patients is BM [10]. After the lung and liver, bone is the third most frequent location for metastatic disease. In developed nations, the incidence of BM disease is over 280,000 cases annually and tends to increase due to a rise in the lifespan of cancer patients [11]. Moreover, BM increases the likelihood of skeletal-related events (SREs) and complications, involving pathologic fracture, significant health, resources utilization, and costs [1,10]. We can improve the BC patient outcome by diagnosing and treating BM before the significant neurologic and functional deficits onset. Proper imaging can help detect bone metastasis early. For example, radionuclide bone scanning is commonly used as the first imaging method to detect skeletal metastases due to its high sensitivity and ability to examine the entire skeleton in a single scan [4]. The follow-up process of a treated BC patient should be regular every three months for the first couple of years, then every six months until the fifth year, and after that, an annual check-up of all systems and symptoms with mammograms. In our case, the patient did not follow up with her GP to ensure her condition's stability after eight years of her BC radical mastectomy, which eventually resulted in pain and discomfort due to the recurrence of the tumor in numerous metastatic lesions in the liver and bone. Within metastatic bone disease management, and considering the necessity of a multidisciplinary team and approach. Orthopedic surgeons must be early-stage involved, not just when patients have pathological fractures. This is critical for cancer patients, particularly those with a limited life expectancy, as prompt intervention, pain management, and enhancing functional status can significantly benefit them [11]. Subtrochanteric femur fractures represent a challenging orthopedic issue, ranging from 10 % to 34 % of all hip fractures. Consequently, numerous factors impact subtrochanteric fractures prognosis as comminution level, bone mineral density, and implant placement and when treating subtrochanteric fractures, orthopedists should consider the suitable implant based on the fracture type and the patient's overall health condition [12]. Palliative treatment notably improves the QoL of cancer mets patients who are bedridden and present with severe symptoms. In addition to providing pain relief, palliative care can also address other patient and their family physical and emotional needs. Malalignment usually happens because conservative treatment for this type of fracture does not provide adequate reduction. Therefore, the majority of surgeons recommend internal fixation. Several internal fixation techniques, such as plate-screw systems and intramedullary nails, are used for this type of fracture. However, the intramedullary nail is currently trendy. Using intramedullary nails to manage femoral subtrochanteric fracture has many benefits, such as closed indirect reduction and fixation through a minimally invasive approach. Consequently, this approach can help minimize the fracture site damage, which is beneficial for the healing and likelihood reduction of internal fixation failure. Additionally, from a biomechanical perspective, an intramedullary nail can provide more stability compared to a plate-screw system. Patients may be able to move sooner after the surgery, which could reduce complications such as pneumonia, urinary tract infections, and deep vein thrombosis [13]. Intramedullary devices are intended to reduce soft-tissue dissection resulting in a lower likelihood of surgical trauma, bleeding loss, infection, and wound complications. Due to its shorter lever arm, the cephalomedullary nail can maintain biomechanical stability when exposed to loading forces. However, it may lead to femoral shaft fractures occurring below the nail in some cases. PFN was Intended to control the drawbacks of the cephalomedullary nail and has been used to treat subtrochanteric and trochanteric fractures, besides preventive nailing [8]. Utilization of extramedullary fixation has certain disadvantages, including prolonged surgical time, increased invasiveness, instability in the medial area, and the likelihood of fracture after the removal of implants [12], even using conventional proximal femoral plates does not yield secure fixation, resulting in complications such as deformity, nonunion, and damage to blood supply [13]. Therefore, the multidisciplinary team identified the subtrochanteric fracture and discussed the surgical options for treatment, following clinical and radiographic guidelines. After a detailed discussion, PFN was the procedure of choice depending on the patient's preferences and tumor prognosis. The technique is frequently reproducible, comparable to the insertion of nails in traumatic fractures treatment [14]. We recorded no postoperative complications and the patient was discharged a day after the surgery. We radiographically identified the success of surgery, with a regular eight monthly follow-up by visits and phone reassuring. The patient is now well-rehabilitated and satisfied after improving her skeletal ability and QoL, despite the metastatic lesions and end-stage conditions. In conclusion, we highlight the surgical decision consequences for a patient with end-stage cancer, as it may put their life at risk or improve their QoL, likewise, the patient in this report. And we also recommend that every patient with cancer is followed up even after complete recovery to avoid complications of metastasis, and the operation is performed by a qualified specialist because there was difficulty in placement PFN due to metastases.

## Consent for publication

Written informed consent was obtained from the patient for the publication of this case report and any accompanying images. A copy of the written consent is available for review by the Editor-in-Chief of this journal.

## Ethical approval

This study is exempt from ethical approval in our institution (Aleppo University Hospital, Faculty of Medicine, University of Aleppo, Aleppo, Syria).

## Funding

This research did not receive any specific grant from funding agencies in the public, commercial, or not-for-profit sectors.

## CRediT authorship contribution statement

All authors were both involved in the conception and coordination of this report and drafted the manuscript. Additionally, all authors have read and approved the final version.

## Guarantor

Mohamad Ali Farho.

## Registration of research studies

1. Name of the registry: Not applicable in Syria.

2. Unique identifying number or registration ID: Not applicable in Syria.

3. Hyperlink to your specific registration (must be publicly accessible and will be checked): Not applicable in Syria

## Declaration of competing interest

No conflicts of interest.

## Data Availability

All data on which the conclusions of this case report are based are included in this manuscript.
